# Cognitive rehabilitation for reversible and progressive brain injury

**DOI:** 10.4103/0019-5545.44752

**Published:** 2008

**Authors:** Ravi Samuel

**Affiliations:** The Psychotherapy Clinic, 26/1 Arcot Street, T. Nagar, Chennai - 600 017, Tamil Nadu, India

**Keywords:** Brain injury, cognitive impairment and behavior and psychological symptoms in dementia, cognitive rehabilitation, rehabilitation

## Abstract

Cognitive rehabilitation (CR) is a specialized treatment procedure to develop the cognition affected by internal or external injury to the brain. The process of cognitive rehabilitation involves assessment of cognitive functions, goal setting, and applying appropriate cognitive exercises to improve the cognitive function. There are two types of CR: Restorative rehabilitation and Compensatory rehabilitation. The CR therapist will make a comprehensive assessment of the impairment and design appropriate cognitive exercises. Studies on the efficacy of CR for brain damage have shown two extremes; one opinion was CR has a positive effect on the patients cognitive functioning and the other opinion was that CR has no effect on the cognitive functioning. This case study examines the dynamics and relevance of CR in reversible and progressive brain injury. It was observed that in reversible condition CR improves cognition and thereby functional ability. In progressive conditions like Alzheimer's disease (AD), CR improves the cognition marginally and thereby improves functional ability and also reduces Behavioral and Psychological Symptoms in Dementia (BPSD).

## INTRODUCTION

Cognitive rehabilitation (CR) is a specialized treatment procedure to develop the cognition affected by internal or external injury to the brain. This is based on the theory proposed by Luria that the recovery of function can occur through new learned connections established through cognitive retraining exercises.[1] The process of cognitive rehabilitation involves assessment of cognitive functions, goal setting, and applying appropriate cognitive exercise to improve the cognitive function.[2]

There are two types of CR: Restorative rehabilitation and Compensatory rehabilitation.[3] Restorative rehabilitation is to enable the person to develop the lost function through specialized computerized and manual cognitive exercises. Compensatory rehabilitation helps the patient to develop use of aids and tools to overcome the impairment. For example, people with poor memory can have a small slip to write down what they need to remember.

The brain needs specific exercises to enable it to regain the lost function after injury; in case of internal event like stroke or external accident like head Injury. CR is normally done after the patient medically stabilizes.[3] CR is also beneficial for people who have developed their brain functioning in a faulty manner due to poor supervision in learning during childhood.

CR therapist will make a comprehensive assessment of the impairment and select appropriate cognitive exercises. For example if a person suffers from severe memory difficulties, the exercises will start with simple exercises like learning five randomly selected words and then recalling as many times during the day. And with the development of the brain; the difficulty and complexity of the exercises will be increased like asking the patient to learn three new words. Initially CR is done through manual exercises and computerized programme with assistance of the therapist. The therapist will enable the patient to perform exercises in areas in which they have difficulty. After the patient develops confidence over their performance in doing the exercises, they will be encouraged to do cognitive exercises at home.

Studies on the efficacy of CR for brain injury have shown two extremes; one opinion was CR has a positive effect on the patients cognitive functioning[4,5] and the other opinion was that CR has no effect on the Cognitive Functioning.[6,7] This case study examines the dynamics and relevance of CR in reversible and progressive brain injury.

### Case 1

Mr. S aged 39 years, working as manager of a multi national company was referred by a Psychiatrist for CR. He was a heavy smoker and also had the habit of drinking alcohol occasionally. One and half years ago, he had severe myocardial infarction and consequent hypoxia, resulting in severe brain injury, leading to severe cognitive impairment.

#### Assessment

His Mini Mental Status Examination (MMSE) score was 10/30; in orientation, he was not able to give the date, day, month, house number, street and pin code, in memory recall he was able to recall only one word and in Language and Praxia he was not able to name objects and construct diagrams. On clinical evaluation, he had very severe memory impairment; he was not able to remember what happened five minutes ago, he did not know the names of his family members and friends, the company he worked, etc. He had poor orientation towards month, date, day, year, place, and time of the day. Though he was a postgraduate he was not able to read and write any language. His new learning ability was absent. He was dependent on his spouse to perform his Activities of Daily Living (ADL) and was always found clinging to her. He was not able to plan and execute any ADL on his own. Diagnosis: The Psychiatrist did not find any psychiatric disorder so prescribed Aricep 10 mg to improve his cognition and referred him for CR.

#### Cognitive rehabilitation

Cognitive rehabilitation was done in his residence for forty minutes, twice a week for six months period. In the beginning of CR therapy, the focus was on memory and orientation. Since his memory was severely affected, the exercises were towards recall of major life events, work assignments and social events. On the basis of restorative principle to facilitate repetitive learning, a big chart with date, day, month, and year was given to him and was asked to inform the details to the carer thrice daily. The therapist during the sessions taught him to use a calendar to find the details, he was made to strike out the past month and date. This enabled him to use compensatory techniques instead of pleading ignorance and not incorporating the day and month concept in his thinking. To enable him to improve his memory towards past life events, information was gathered from the family members and detailed account of his past was repeatedly narrated to him by the therapist. After two months of cognitive exercises, there was an improvement in his memory in terms of remembering five life events.

Considering the improvement, his cognitive exercises were increased in complexity to enable higher level of stimulation to the brain. The memory exercises involved recalling names of friends and family members, his orientation towards the date and day was poor, so an orientation card was given to him with the year, date, month, and day. Unfortunately, he never bothered to carry it with him or tried to register the information. So the carer was requested to repeatedly use details of day, month, and date in her conversation.

After six months of providing details of his life events and facilitated recall exercises of life events, he was able to give additional details of the events narrated to him. Repeated exercises brought considerable improvement in his memory; he was able to remember the name of the therapist, the name of the hospital, the nature of the therapy and why he had to do the exercises repeatedly. The carer reported that as his cognitive functions developed improvements were noticed in his behavior; his expressions of anger were replaced by courtesy and politeness.

#### Post assessment

The MMSE improved to 15/30; in orientation, he was able to give the date, day, month, and year, he was able to recall one word in the memory test. He was able to identify people whom he meets everyday. Patient was also observed to share social courtesies with visitors. He was able to independently engage himself with television or CD player without clinging to his wife.

### Case 2

Mr. N aged 68 years, with history of diabetes mellitus was brought by his wife for the complaint of progressive memory loss, word finding difficulty, inability to read and write and searching for belongings all the time.

#### Assessment

His MMSE score was 15/30; he had total disorientation, was able to repeat only two words immediately and was not able to remember any words in memory recall exercise. In word fluency test, he was able to recollect five names of animals in one minute, which was suggestive of poverty of words to facilitate conversations. On clinical behavior assessment, he was found to be withdrawn, avoided talking to his family members, kept searching for his misplaced belongings all the time. Patient's wife was found to be extremely critical of the patient, which made him all the more nervous about his performance.

#### Diagnosis

Mr. N had insidious onset of the current illness three years ago, after detailed evaluation the Psychiatrist diagnosed Alzheimer's disease (AD) with co-morbid depressive features. He prescribed rivastigmine 3 mg twice daily and decided not to start pharmacotherapy for his depressive features.

#### Cognitive rehabilitation

Cognitive rehabilitation exercise was provided in out patient facility once a week for forty minutes each session for six months period. Considering the progressive decline in AD very limited cognitive rehabilitation goals were set to improve his orientation, memory and word recall.

To improve his speech and memory Mr. N was encouraged to speak on favorite topics of his choice. He was very much interested in religion and music, he felt encouraged to talk more and more on the subject. To improve word recall the therapist made the patient repeat after him 20 commonly used words in everyday life. Though he repeated the words, he was not able to remember and incorporate it in his speech. So the exercise was modified from words to sentences and were repeated over two week's time every day. After repeated learning for two weeks, he was able to use some of the words in his conversation.

To help him to be oriented towards the day and month, based on restorative principle he was made to relearn the day and month, but even after three weeks of repeated learning patient was not able to register the month and day. So the orientation was modified towards his immediate surroundings. In the house, he was requested to name the objects in the room. He had difficulty in naming things which he does not use like computer, printer, but was able to name familiar objects. Instead of leaving him on his own in the house, a schedule was made incorporating activities like watching television, talking with his wife and daughter.

Patient's wife was counseled to talk about her frustrations during therapy sessions and not be irritable and critical of the patient as it will inhibit his performance.

#### Post assessment

His MMSE score on post assessment was 13/30 after six months. After six months of twice a week cognitive rehabilitation there was no improvement in his score, instead there was deterioration in his orientation, immediate recall and naming objects, patient showed orientation to rooms in the house, was able to keep all his belongings in one place which stopped his endless searching habit. He also helped his wife by participating in cooking and his communication with family members improved.

## CONCLUSION

The present case studies, observed improvement in the cognitive functioning as reported by Gatz *et al*. and Spector.[1,2] In progressive conditions like AD, though CR does not reverse the cognitive impairment, marginally improving the cognition for a short period can reduce Behavioural and Psychological Symptoms in Dementia (BPSD). Also short term developments are also important to maintain the well-being of the patient, particularly in progressive degenerative disorder like dementia.[8]

In the learning process, the therapist compensating by his performance for the patient's cognitive impairment can lead to faster learning. Reading loud the detailed account of incidents, the sentences commonly used, enabled the patients to relearn the material. Similarly, for orientation, the therapist saying once in every ten minutes the day, date, month, year, and season facilitates orientation.

Life circumstances can influence development of cognitive symptoms and that may in turn lead to BPSD. As in Case II, because of critical spouse, patient reduced his speech got isolated, withdrawn, and asocial. And that presumably lead to under usage of language faculty in the brain, which lead to word finding difficulty and poor sentence construction. Modifying life circumstances by counselling the patient and carer, we can avoid ‘induced’ cognitive impairment and BPSD.
